# Difficult Airway Management: From the Power of Prediction to the Art of Management

**DOI:** 10.1213/ANE.0000000000007202

**Published:** 2025-01-10

**Authors:** Thomas Heidegger, Jaideep J. Pandit

**Affiliations:** From the * Department of Anaesthesia, Spital Grabs, Grabs, Switzerland; † Department of Anaesthesia, Bern University Hospital, University of Bern, Bern, Switzerland; ‡ Nuffield Department of Anaesthetics, University of Oxford, Oxford, United Kingdom.

**See Article, page 295**

There are 2 recent reasonably large scale studies that investigate decision-making and processes used in the anticipated difficult airway. In this issue of *Anesthesia & Analgesia*, Yang et al^1^ report on a convenience sample of 1245 patients predicted to be difficult in a single US center from 2009 to 2014. An earlier-published study by Cumberworth et al^2^ (but one that related to a later cohort of patients in 2018) was set in 4 UK hospitals over 1 calendar year, capturing an estimated 61,000 patients of which 4100 (6.7%) were predicted difficult and of which 17 resulted in serious airway complications (specified as: brain damage; emergency (unplanned) front of neck access (FONA); unanticipated intensive care unit (ICU) admission due to airway complications; prolongation of ICU stay (due to airway management complications in theater for patients already in ICU); need for active airway management in the postanaesthesia care unit (PACU); failed airway management leading to cancelation of surgery on the day). The core data and results of these studies are summarized in the Figure and Table 1.

## THE POWER OF PREDICTION

The 2 studies have several differences, but one important feature in common; namely, that practitioners predicted airway difficulty. The utility of difficult airway prediction is something that has been hotly debated over the years, with one extreme view being that it is a “pointless ritual.”^3^ This nihilistic conclusion is based on the reality that no single clinical bedside test, or even combination of tests, yields specificity values for prediction (or receiver operating curve, ROC, results) that are regarded by convention as acceptable for most clinical tests.^4^ For example, the best positive predictive values reported remain doggedly at ~35% (meaning that, of every 3 patients whose airway is predicted difficult, just one will prove so once airway management is commenced). Furthermore, Yentis^3^ argued that because the incidence of the difficult airway was so rare, it was a fundamental principle of statistics that no test would ever be discovered that would satisfy conventional thresholds for satisfactory prediction. He underlined the argument by proposing his YETI test (Yentis test-of-intubation) which simply scored each patient as “easy airway.” Even if on ~1% of cases in which this test would fail and the patient proved difficult, the YETI test would have an impressively good sensitivity of 99% for prediction of the easy airway.^3^

**Table 1.** Summary Proportions Estimated From the Primary Studies for Those Patients Predicted Difficult

	Cumberworth et al^2^	Yang et al^1^
Awake/sedated intubation	2.7%	13%
HFNO	9.5%	^a^
VL	25%	71%
>3 attempts	24%^b^	21%

Abbreviations: HFNO, high-flow nasal oxygen; VL, videolaryngoscopy.

a No HFNO available, but 50% incidence of desaturation.

b This proportion is of those 17 patients with critically adverse outcome.

**Figure.**
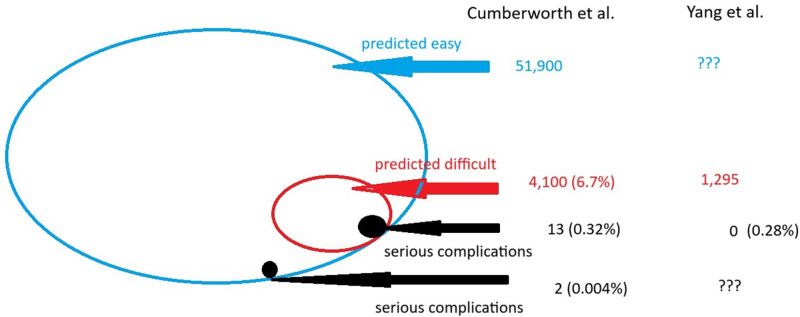
Illustration of the key results of Yang et al^1^ and Cumberworth et al.^2^ The outer blue oval represents the estimated 51,900 predicted easy intubations assessed by Cumberworth et al; combined with the 4100 predicted difficult (red oval) the total population was 56,000. Yang et al did not report all intubations, but only the predicted difficult (red oval: 1295 in Yang et al). Of these predicted difficult, there were 13 serious complications (black circles) in Cumberworth et al (0.32% of the predicted difficult), but none in Yang et al which, applying the upper limit of the 95% binomial confidence interval, in fact yields a similar proportion (0.28%). Cumberworth et al also reported a very small proportion of serious complications in those patients predicted easy.

These criticisms of the predictive ability of anesthesiologists have been overturned by our later reasoning.^5^ This is based on difficult airways being heterogenous: there is nothing really in common with a patient with trismus and one with a large, compressing goiter causing upper airway obstruction. Just as oncologists do not regard all cancers as a single group, or infectious disease doctors do not view malaria as equivalent to urinary tract infection, there is no reason to propose that anesthesiologists should view these 2 classes of patient as equivalent. Nevertheless, both these types of patient are readily predicted by competent practitioners using bedside tests to be difficult (ie, almost 100% positive predictive value); it is just that they are difficult each in their own way. Once we realize this, we move away from nihilism to an active, positive identification of difficulty, or more accurately, of a patient’s airway being “not easy” in a specific way, and this is what alters the statistics. The correct questions to ask are as follows: (a) *in patients whose airway is predicted easy, how likely is failed airway management*? We already know this is very low, say ~1%.^6^ And (b) *in patients whose airway is predicted difficult, how often is that proved correct?* We know this is ~30%.^4^ Therefore, our bedside tests (and it does not really matter which ones we prefer) are 30 times as likely to make a correct prediction of difficulty, which is in fact a very powerful test.

## CLINICAL MANAGEMENT IN THE STUDIES

In turn, prediction should lead to tailored management. Again, the language of the “airway management community” writ large can be imprecise. The “difficult airway” encompasses several possible more discrete difficulties within management: for example, difficult mask ventilation, difficult supraglottic airway (SGA) insertion, difficult laryngoscopy, difficult oxygenation, etc. We collectively often refer to an SGA as a “rescue device.”^7,8^ Yet it cannot rescue any patient with intractable trismus. We often refer to fibreoptic intubation as a “gold standard” (witness the opening lines of the UK’s Difficult Airway Society guidelines^9^): yet is no standard at all in a patient with trismus and hemorrhagic nasal polyps. And so on. In other words, we should not aspire to a universal, default or standard management algorithm when we predict a “difficult airway.” Rather, we should develop an airway management plan (and that includes Plan A, B, C, etc) that is specific to the airway challenge faced.

The study of Cumberworth et al^2^ showed the price of failing to do just that. In that study, the incidence of in-operating room serious airway complications after prediction of an easy airway was just 1 in 26,000 (0.004%). Strikingly, the management plans did not materially differ in that group whose airways were predicted difficult, with similar (low) rates of using fibreoptic intubation, videolaryngoscopy or high-flow nasal oxygen (HFNO). The result was a serious airway complication rate in this group of 1 in 315 (0.32%). The message is clear. If we predict patients as difficult but then manage them as if easy, we increase their risk of serious complications ~83-fold. This is despite using the failed intubation guidelines^9^ as a “safety net.”^10^

The perspective of Yang et al’s study is only from that of those patients already predicted difficult; that is, we do not know the management or fate of those predicted easy. Nevertheless, the headline message appears to be that overall management was “successful” with no serious complications or abandonment of surgery. That said, applying 95% confidence intervals to zero numerators in fact yields a “worst expected” incidence of serious complications in the predicted difficult group of 0.28%; similar to the Cumberworth study.^11^ There was significantly higher use of awake/sedated fibreoptic intubation (13%) and videolayryngoscopy (71%) than in Cumberworth et al’s study (25% in those with complications, 3% overall). High-flow nasal oxygenation had not then been described, and the high incidence of hypoxia during awake/sedated intubation (50%) underlines not only possible oversedation but also suboptimal oxygenation during the procedure.

However, we should not be misled by the apparent “success” in the data of Yang et al. This is something they stress in their conclusions: such success rates do not fully describe the true risks. There are clearly shortcomings in management that mean with a larger cohort (note that Cumberworth et al’s difficult patient pool was 4 times as large), serious complications may have been seen. The hints that this may be the case include the high incidence of desaturation referred to above not only in the awake-managed group, but also in those in whom anesthesia was induced (30%), as well as cardiovascular instability (21% overall).

Moreover, there were serious deviation from published guidelines. Notably, in one-fifth (21%) of all cases there were 3 or more attempts at intubation. By definition of most all airway management guidelines, 3 attempts (plus possibly one more by a second practitioner called to assist) is in fact failure of airway management.^12,13^ The real, important result of Yang et al’s study is that with the approach described in patients predicted difficult, one-fifth of attempted intubations fail. The corollary is that a different approach is needed for this group of patients.

## WHAT IS “DIFFICULT?”

In common parlance, difficulty implies that some nonstandard approach is needed to overcome the challenge. This applies as much to mathematics (where novel methods of analysis might be used) as it does to skiing (where techniques used on gentle beginner runs will not work on steep off-piste slopes). Therefore a “difficult airway” means 1 in which were standard methods to be used, a failed intubation is likely. This is notwithstanding the notion of a “physiologically” vs “anatomically” difficult airway.^14^ It follows that we should debate what is ’standard management’, but generally it can be assumed to be induction of anesthesia followed by an SGA (without prior paralysis) or direct laryngoscopy for tracheal intubation (after neuromuscular blockade). In the latter, this is notwithstanding the promotion of videolaryngoscopy as now a standard technique.^15,16^

In this context, “nonstandard” includes any one or more of the following (in no particular order): dedicated oxygenation throughout the procedure (such as HFNO^17^); awake or sedated intubation (fibreoptic); videolaryngoscopy with hyperangulated blade; rapidly acting neuromuscular blockade^18^; awake tracheostomy; assembling assistance with a team.^19^

## CONCLUSIONS: TOWARD THE PROPER ART OF MANAGEMENT

The key decision point, before the start of any management and cited by Yang et al and enshrined in the American Society of Anesthesiologists’ 2022 (predicted) Difficult Airway algorithm^13,20^ is whether to induce anesthesia or not before airway management. By their definition, with which we strongly concur, there is no logic to inducing general anesthesia before securing the airway in a patient predicted to be difficult. Since all (1239) the patients in Yang et al’s group were predicted difficult, they should therefore all have undergone awake/sedated intubation: yet, only ~13% (166) did so. Moreover, a high proportion needed multiple attempts, endured desaturation and cardiovascular instability and for 3 patients, Plan B seemingly and paradoxically involved induction of anesthesia. It is assumed that awake/sedated techniques will better maintain oxygenation, but Yang et al^21^ have separately reported desaturation in 26% of cases. However, it is not clear yet if desaturation is an inevitable consequence of the technique, or if it should be regarded as process failure, since Heidegger et al^22^ reported only 2 desaturations in 1612 patients (0.12%). In these generalizations, we recognize acceptable modifications to the principle of “securing the airway before anesthesia induction,” such as the protocol used in St Gallen, Switzerland, where the patient is induced just before actual passage of tube into trachea, the fibrescopy having been otherwise conducted awake or sedated, and advanced into the trachea.^22^ We also recognize that “awake” and “sedation” in this context has a range of meanings, as previously discussed,^23^ but all of these fall well short of any definition of “general anesthesia.”

It is not clear why so many attempts were permitted, both for this awake-managed and anesthesia-induced group in the Yang et al study, and whether peer- or surgical-pressure was responsible. Multiple attempts >3 may be justified in a given case (eg, if mask ventilation in between attempts remains successful). However, practitioners should bear in mind that should an adverse outcome result, later enquiry of medicolegal analysis might be extremely critical and interpret this as reckless persistence. The only relief is that Yang et al’s study is old; a feature of many large airway management audits, including that of Cumberworth et al, and it is hoped practice may have improved. Both these studies warrant repeating.

The 4th National Audit Project (NAP4) of the United Kingdom (another airway study that took almost 4 years to be finalized) bemoaned the low incidence of techniques such as fibreoptic intubation, and recognized a disconnect between airway prediction (which was under-used, largely due to the previous nihilistic approach), and management.^24^ The accompanying editorial noted that the clinical case which had triggered this national audit had undergone a “routine” anesthetic some weeks before his fatal airway management failure.^25^ It is believed that he had undergone standard induction and SGA successfully (the team there had “gotten away with it”), but when this was repeated at the second hospital (despite several strong signs of difficult airway) catastrophe followed.

Each of the points we make and the techniques and process we have discussed needs to be the basis of ongoing research but the state of evidence leads to conclusions which seem to us very clear (Table 2). First, use bedside tests to make a binary prediction as to whether the airway is easy or difficult. If difficult, then define the cause of difficulty and tailor the management in a way that (a) reliably maintains oxygenation and (b) has a high chance of success. Third, maintain training in advanced and specialized techniques (eg, HFNO, videolaryngoscopy, fibreoptic intubation, emergency FONA). Fourth, team train in human factors. In the elective setting, limit all attempts to no more than 3, even if that means postponing the case. Finally, use the *post hoc* findings to “close the loop” on the initial prediction: if the airway was judged “easy” but proved difficult, reflect on why as a self- and team-learning exercise. This also applies if the airway was judged “difficult” but later proved “easy”; however, it is possible that in such cases, specialized management techniques were justified, given the clinical signs.

**Table 2.** Key Ingredients For Safe Difficult Airway Management

•Airway assessment: is the airway “easy” or “difficult?” (binary approach)
•If difficult: define the underlying cause (eg, difficult mask ventilation; difficult oxygenation^a^; upper airway obstruction^b^; lower airway pathology^c^)
•Tailor the management to the underlying cause in a way that (a) reliably maintains oxygenation and (b) has a high chance of success^d^
•Maintain personal training in advanced and specialized techniques (eg, HFNO, videolaryngoscopy, fibreoptic intubation, emergency FONA).
•Team train in human factors and communication
•For elective cases, limit all attempts to <4^e^; postpone case if >4
•‘Close the loop’ on initial prediction: if mis-predicted as easy when difficult, reflect on clinical signs and thresholds applied; or affirm that clinical signs justified the specialized techniques, even if airway proved easy

Abbreviations: FONA, front of neck access; HFNO, high-flow nasal oxygenation.

a This includes obesity.

b For example, a compressing goiter.

c For example, chest wall deformity, lung disease.

d Note that a single method (eg, fibreoptic intubation) may not be applicable to all.

e Guidelines recommend a maximum of 3 by 1 practitioner, and a fourth attempt by another.

Even though perfect airway prediction is impossible, the clinical methods we have are sufficiently precise as to guide safe practice by helping create tailored plans for those predicted difficult. Even those airways identified as “easy” where the risk of actual difficulty is very low benefit from thinking about possible Plan B, C, etc. Coupled with maintaining rigorous training and reflection, we can transform difficult airway management into a more predictable success.

## DISCLOSURES

**Conflicts of Interest:** J. J. Pandit is Editor-in-Chief of *Anesthesia & Analgesia*; he was not involved in the handling of this article. No other authors declared Conflicts of Interest. **Funding:** None. **This manuscript was handled by:** Narasimhan Jagannathan, MD, MBA.
